# Role of CT in Pyelonephritis: A Comprehensive Pictorial Review

**DOI:** 10.7759/cureus.88666

**Published:** 2025-07-24

**Authors:** Sundararajan Srinivasan, Arun Biradar, BH Parameshwar Keerthi, Maghiben Mohan

**Affiliations:** 1 Department of Radiology, Meenakshi Medical College Hospital and Research Institute, Meenakshi Academy of Higher Education and Research (Deemed to be University), Kanchipuram, IND

**Keywords:** chronic pyelonephritis, computed tomography, emergency, emphysematous pyelonephritis, pyelonephritis, renal abscess, xanthogranulomatous pyelonephritis

## Abstract

Pyelonephritis in its various forms represents a urologic emergency that can lead to significant morbidity and requires prompt diagnosis and appropriate management. While clinical assessment and laboratory findings remain the cornerstone for diagnosis, imaging studies play a crucial role in evaluating the extent of infection, identifying complications, and guiding therapeutic interventions. Computed tomography (CT) has emerged as the imaging modality of choice for evaluating the severity of patients with pyelonephritis, offering superior sensitivity and specificity compared to other imaging modalities.Through illustrative cases and a systematic approach to image interpretation, this pictorial essay aims to comprehensively review the CT imaging features of various forms of pyelonephritis, highlighting characteristic imaging features of each entity, potential complications, and key differential diagnoses that help in differentiating them from their mimics, thus enhancing radiologists' and clinicians' ability to recognize the diverse CT appearances of this common but potentially serious infection.

## Introduction and background

Urinary tract infections are among the most common clinically encountered infections [1]. Pyelonephritis is defined as an infection of the renal parenchyma and collecting system, typically ascending from a lower urinary tract infection. While uncomplicated cases of pyelonephritis generally respond well to antibiotic therapy without the need for imaging, certain patient populations, including those with diabetes, immunocompromised states, history of urolithiasis, or previous renal surgery, require imaging evaluation to assess disease severity and detect complications [2,3].

Computed tomography (CT) has established itself as a preferred modality for evaluating pyelonephritis, with its excellent spatial resolution and ability to assess both parenchymal and perinephric changes [4]. With its reported sensitivity and specificity in the diagnosis of acute pyelonephritis ranging from 86% to 98% and 87% to 97%, respectively, CT can effectively detect subtle abnormalities that might be overlooked by alternative imaging techniques [5]. While not routinely indicated for uncomplicated urinary tract infections, contrast-enhanced CT provides valuable insights for high-risk patients by defining disease extent and identifying potential complications.

## Review

CT imaging protocol

The optimal CT protocol for evaluating suspected pyelonephritis typically includes three phases: non-contrast, nephrographic, and excretory phases. The non-contrast phase serves as a baseline for evaluating enhancement patterns and is also essential for detecting calcifications, calculi, gas, and hemorrhage [6]. The nephrographic phase is acquired usually 80-100 seconds after intravenous contrast administration and is most sensitive for parenchymal abnormalities. The reported accuracy of the nephrographic phase for acute pyelonephritis diagnosis is 90%-92% [6]. The nephrographic phase is also known as the nephrogenic phase. The excretory phase is acquired 5-10 minutes after contrast administration and evaluates contrast excretion and urinary tract patency, thus being of value in suspected urinary obstruction [6,7].

The estimated effective dose ranges from 8 to 15 mSv for a complete CT urography examination, with specific adjustments based on patient factors and clinical indications [8]. In certain clinical cases, multiple follow-up CT examinations may become essential to track treatment progress, which underscores the need to develop low-dose CT protocols to minimize cumulative radiation exposure to patients without sacrificing diagnostic quality [9]. Radiologists must emphasize evaluating CT imaging findings in conjunction with patients' clinical presentations and relevant laboratory findings for optimal diagnostic accuracy [2,5].

Pyelonephritis types and their CT imaging features

Uncomplicated Acute Pyelonephritis

Acute pyelonephritis typically manifests with fever, flank pain, and laboratory markers of infection. The infectious process typically begins with the colonization of the lower urinary tract and ascends to the renal pelvis and parenchyma, commonly facilitated by conditions such as vesicoureteral reflux or urinary obstruction [10]. The predominant causative pathogens are gram-negative bacteria, with *Escherichia coli* responsible for the majority of cases, followed by other organisms such as *Proteus*, *Klebsiella*, and *Enterococcus* species [11].

**Figure 1:**
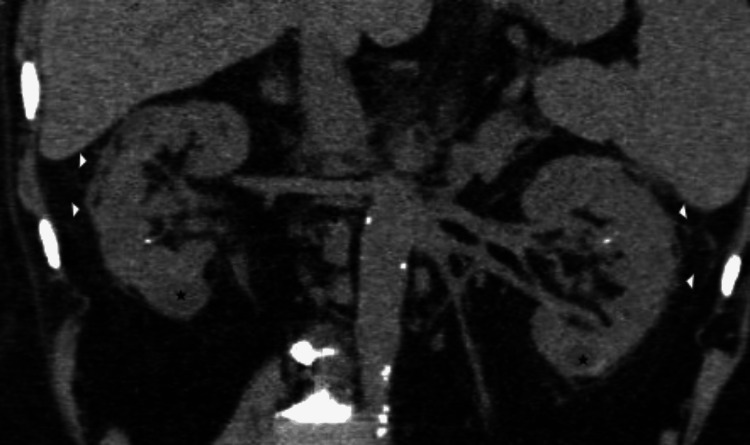
Acute pyelonephritis on non-contrast CT. A 43-year-old woman presenting with an acute history of fever and abdominal pain was diagnosed with acute pyelonephritis. Coronal reformatted non-contrast CT of the abdomen image shows bilateral enlarged kidneys with perinephric fat stranding (arrowheads) around both kidneys. A few renal calculi and well-defined hypodense cortical cysts (black asterisks) are also seen in both kidneys. These images are from the hospital and have not been published elsewhere. CT: computed tomography

On CT imaging, it can present in either focal or diffuse patterns with findings ranging from subtle parenchymal alterations to obvious inflammatory changes. Non-contrast CT examinations may show the diffuse or focal enlargement of the affected kidney relative to the contralateral side, inflammatory stranding in the perinephric fat, and reactive thickening of the renal fascia (see Figure 1) [5].

**Figure 2:**
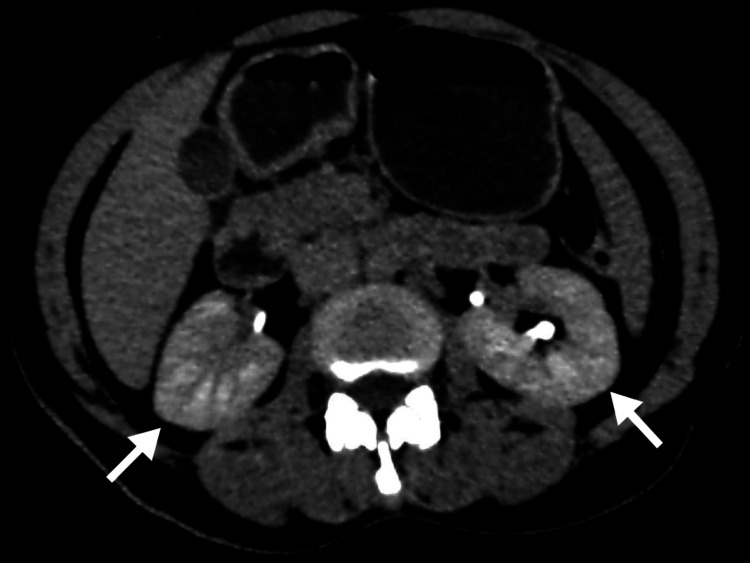
Acute pyelonephritis with striated nephrogram on contrast-enhanced CT. A 55-year-old woman with complaints of right loin pain was diagnosed with acute pyelonephritis. Contrast-enhanced axial CT of the abdomen image of the patient shows a striated nephrogram (arrows). These images are from the hospital and have not been published elsewhere. CT: computed tomography

Contrast-enhanced studies provide additional valuable information, revealing wedge-shaped regions of decreased enhancement that represent focal areas of inflammation, optimally visualized during the nephrographic phase [12]. Other findings may include diminished corticomedullary differentiation and striated nephrogram (see Figure 2), which appears as linear alternating bands of increased and decreased density in the renal medulla, resulting from tubular obstruction and interstitial edema [7].

It should be noted that immunocompromised patients may demonstrate a reduced inflammatory response, resulting in less prominent imaging findings despite significant infection [3]. Bilateral kidney involvement is observed in approximately 20% of cases and may indicate hematogenous spread rather than ascending infection [10].

Differential diagnosis for acute pyelonephritis: The differential diagnosis for acute pyelonephritis encompasses several conditions with overlapping imaging features, which include renal infarction, renal lymphoma, and acute interstitial nephritis. Renal infarction can be distinguished by its sharply demarcated wedge-shaped areas of hypoattenuation with preserved subcapsular cortical enhancement (the "cortical rim sign") [13]. These areas do not improve with antibiotic treatment and are frequently associated with underlying vascular pathology or embolic events. Renal lymphoma may appear as multiple poorly enhancing hypodense lesions or diffuse parenchymal infiltration on contrast studies; however, it typically lacks perinephric inflammatory changes and is often accompanied by retroperitoneal lymphadenopathy [14]. Acute interstitial nephritis generally demonstrates a more diffuse pattern with bilateral kidney involvement, is commonly linked to medication exposure or systemic disorders, and does not display the wedge-shaped abnormalities characteristic of pyelonephritis [15]. Clinical history and laboratory findings are essential for accurate differentiation among these entities.

Emphysematous Pyelonephritis (EPN)

Emphysematous pyelonephritis (EPN) represents a severe, necrotizing form of infection characterized by gas formation within the renal parenchyma, collecting system, or perinephric tissues. Multiple factors contribute to the pathophysiology; however, it predominantly affects patients with diabetes mellitus (approximately 90% of cases) and is associated with glucose fermentation by gas-forming organisms, most commonly *E. coli* and *Klebsiella pneumoniae* [16].

**Figure 3:**
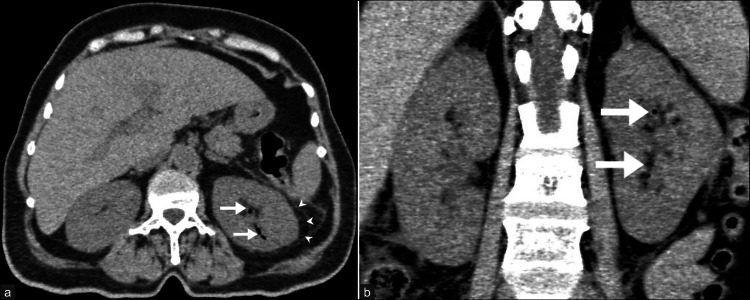
Early emphysematous pyelonephritis. A 62-year-old woman with a history of fever, vomiting, and abdominal pain was diagnosed with early emphysematous pyelonephritis. (a) Non-contrast axial CT of the abdomen image shows a few air foci (arrows) in the parenchyma and pelvicalyceal system of the left kidney, along with minimal perinephric fat stranding. (b) Coronal reformatted image of the left kidney shows the extent of air foci. These images are from the hospital and have not been published elsewhere. CT: computed tomography

**Figure 4:**
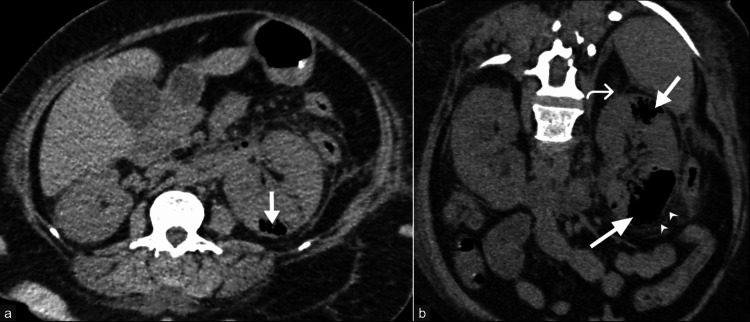
Class 3A emphysematous pyelonephritis with perinephric extension. A 50-year-old woman was diagnosed as Class 3A emphysematous pyelonephritis (as per the Huang and Tseng classification [9]). (a) Non-contrast axial CT KUB image shows multiple low-attenuation air foci (arrows) within the parenchyma and perinephric space of the left kidney, along with the thickening of renal fascia. (b) Coronal reformatted image shows large air pockets in the upper and lower poles of the left kidney with parenchymal destruction and extensive perinephric fat stranding and fluid (arrowheads). These images are from the hospital and have not been published elsewhere. CT, computed tomography; KUB, kidney, ureter, and bladder

**Figure 5:**
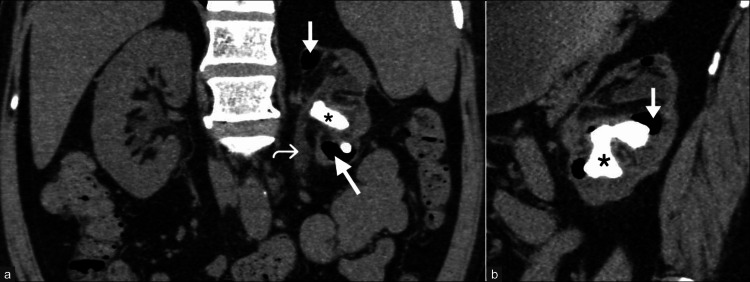
Emphysematous pyelonephritis with staghorn calculus and urinary obstruction. A 52-year-old male patient presenting with complaints of left flank pain and a thin stream of urine was diagnosed with emphysematous pyelonephritis. (a) Coronal reformatted image of non-contrast CT of the abdomen shows a relatively small left kidney with multiple pockets of air (arrows) in the renal parenchyma and pelvicalyceal system, a hyperdense calculus (asterisk) in the pelvicalyceal system, hydronephrosis, dilated ureter (curved arrow), and perinephric fat stranding. (b) Sagittal reformatted CT image demonstrates the calculus to be a "staghorn" calculus with adjacent emphysematous changes (arrow). These images are from the hospital and have not been published elsewhere. CT: computed tomography

CT is the imaging modality of choice for diagnosing EPN, with the distinctive finding being gas collections appearing as low-attenuation foci (approximately -1000 HU) within the renal parenchyma, collecting system, or surrounding tissues (see Figures 3-5) [17]. Associated findings include renal enlargement, the absence of contrast enhancement in affected areas, perinephric fluid collections, and the thickening of Gerota's fascia [14].

Early CT diagnosis is critical for improving outcomes in this life-threatening condition, with findings often categorized according to established classification systems that stratify disease severity and prognosis. The Huang and Tseng classification categorizes EPN into different classes: Class 1 involves gas limited to the collecting system (emphysematous pyelitis), Class 2 shows gas in renal parenchyma without extension to perinephric spaces, Class 3A demonstrates the extension of gas to the perinephric space, Class 3B shows the extension of gas to pararenal space and the involvement of adjacent structures, and Class 4 represents bilateral emphysematous pyelonephritis or the involvement of a solitary kidney [16]. Alternatively, the Wan et al. classification divides EPN into two types: Type I demonstrates renal necrosis with the presence of gas but no fluid, while Type II shows the presence of gas and fluid in the renal parenchyma, collecting system, or perinephric space [17]. The CT-based classification of EPN directly influences management decisions, with higher classes often requiring more aggressive intervention, including possible nephrectomy in refractory cases [16].

Differential diagnosis for emphysematous pyelonephritis: The differential diagnosis for emphysematous pyelonephritis comprises other gas-forming conditions of the urinary tract, such as emphysematous pyelitis, gas-forming renal abscesses, and pneumaturia from fistulous connections. These conditions can be differentiated based on their distinct imaging characteristics. Emphysematous pyelitis is distinguished by gas confined to the collecting system without parenchymal involvement and typically carries a more favorable prognosis compared to emphysematous pyelonephritis [14]. Gas-forming renal abscesses appear as more focal gas collections contained within a defined abscess cavity surrounded by an enhancing rim [18]. Pneumaturia resulting from a fistulous connection presents with gas in the collecting system without parenchymal gas involvement but demonstrates evidence of an abnormal communication between the urinary tract and adjacent hollow organs [15].

Xanthogranulomatous Pyelonephritis (XGP)

Xanthogranulomatous pyelonephritis (XGP) is an uncommon, chronic inflammatory form of pyelonephritis characterized by the destruction of renal parenchyma and replacement with lipid-laden macrophages, known as xanthoma cells [19]. It typically develops in the setting of chronic urinary obstruction, most frequently due to nephrolithiasis, and is commonly associated with infections by *Proteus mirabilis* or *Escherichia coli*, leading to inflammatory changes in surrounding tissues [20]. XGP can manifest as either diffuse or focal forms, with the diffuse form being much more common [21].

**Figure 6:**
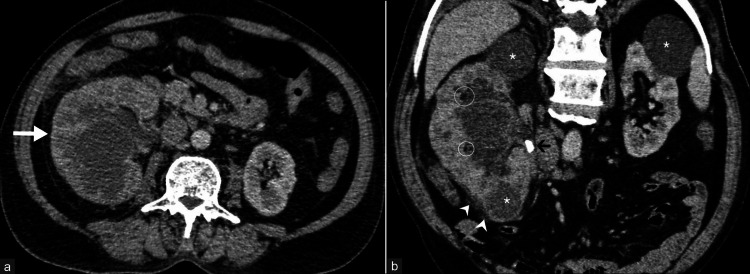
Xanthogranulomatous pyelonephritis with architectural distortion. A case of xanthogranulomatous pyelonephritis was diagnosed in a 70-year-old man. (a) Nephrographic phase axial CT urogram image shows grossly enlarged right kidney with gross pelvicalyceal dilatation. (b) Coronal reformatted image in nephrographic phase shows the architectural distortion of the right renal parenchyma and areas with fat attenuation foci (circles) with reduced enhancement compared to the left kidney, indicating declining renal function. Perinephric fat stranding (arrowheads) and the thickening of the right renal fascia are noted. An obstructive calculus (black arrow) is seen in the right pelviureteric junction. Multiple well-defined non-enhancing hypodense cortical cysts are seen in both kidneys (white asterisks). These images are from the hospital and have not been published elsewhere. CT: computed tomography

**Figure 7:**
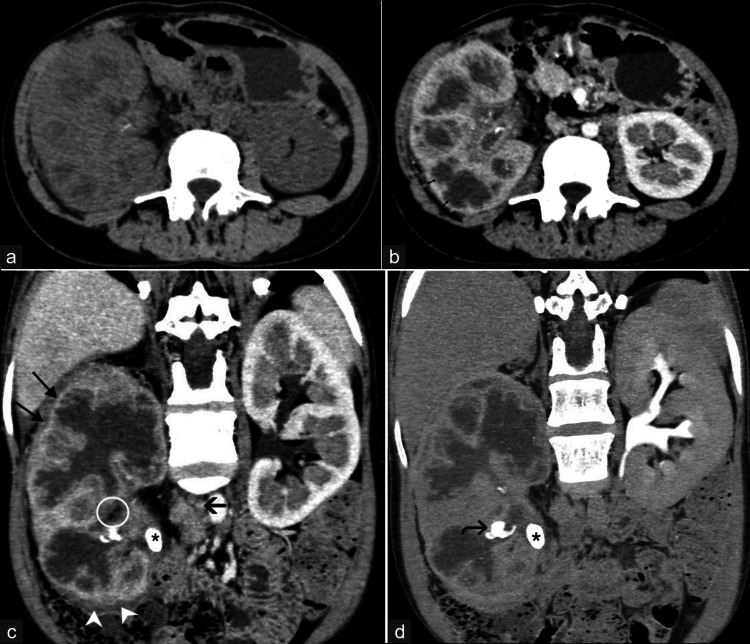
Xanthogranulomatous pyelonephritis demonstrating classic "bear paw" sign. A 32-year-old female patient complaining of right-sided abdominal pain was diagnosed with xanthogranulomatous pyelonephritis. (a) Non-contrast axial CT image shows an enlarged right kidney with edematous cortex and hydronephrosis. (b) Nephrographic phase axial CT urogram image reveals minimally enhancing heterogeneous, thinned-out right renal parenchyma (double-headed arrows) and grossly dilated pelvicalyceal system with "bear paw" sign. (c) Nephrographic phase coronal reformatted image shows moderate perinephric fat stranding (long black arrows), the thickening of renal fascia, minimal perinephric fluid (arrowhead), and a few enlarged retroperitoneal lymph nodes (short black arrow). Areas with few fat density foci (circle) are seen in the renal parenchyma. An obstructive calculus (black asterisk) is seen in the pelviureteric junction. (d) Coronal reformatted image of a four-hour-delayed phase shows no contrast opacification in the collecting system and ureter on the right side, indicating reduced function of the right kidney. A few renal calculi (black curved arrow) are also seen. These images are from the hospital and have not been published elsewhere. CT: computed tomography

The diffuse form typically presents on CT with an enlarged kidney showing the loss of normal architecture and poor or absent contrast enhancement, indicative of poor renal function (Figure 6). Renal calculi are present in 70%-80% of cases, often as central staghorn or branching calculi in the renal pelvis [19]. Multiple hypodense areas representing dilated calyces with debris may be seen, along with the thinning of the residual renal parenchyma. In contrast studies, enhancing rims can be observed around these areas, creating the characteristic "bear paw" sign (Figure 7) [20]. The inflammatory process frequently extends beyond the kidney into the perinephric and pararenal spaces, psoas muscle, and abdominal wall.

In focal XGP, CT may show a localized inflammatory mass with an associated calculus in the adjacent calyx and a lesser degree of parenchymal destruction. This localized mass-like appearance may clinically and radiologically resemble renal cell carcinoma, presenting a significant diagnostic challenge [21].

Complications such as fistula formation to adjacent organs, including the skin, pleural space, or bowel (particularly the colon), should be carefully evaluated in a case of XGP [21].

Differential diagnosis for xanthogranulomatous pyelonephritis: The differential diagnosis of xanthogranulomatous pyelonephritis encompasses several conditions with similar radiological features, specifically renal cell carcinoma, renal tuberculosis, and renal abscess. Renal cell carcinoma typically exhibits more heterogeneous enhancement patterns but generally lacks the central calcifications seen in XGP. Malignancy rarely causes urinary obstruction, and patients do not typically have a history of recurrent urinary tract infections [22]. Renal tuberculosis is characterized by the presence of multiple smaller calcifications rather than staghorn calculi, accompanied by ureteral strictures and bladder contraction. Inflammatory changes in the perinephric region are generally minimal [14]. Renal abscess typically has a more acute clinical presentation and demonstrates a well-circumscribed fluid collection without the distinctive "bear paw" pattern. Calculi are often absent [4]. In challenging cases, clinical correlation and sometimes histopathological assessment may become essential for definitive diagnosis.

Chronic Pyelonephritis

Chronic pyelonephritis develops from recurrent or inadequately treated episodes of acute pyelonephritis, frequently in the setting of vesicoureteral reflux or persistent urinary obstruction, with the recurrent inflammatory processes leading to progressive scarring, fibrosis, and calyceal deformities [22,23].

**Figure 8:**
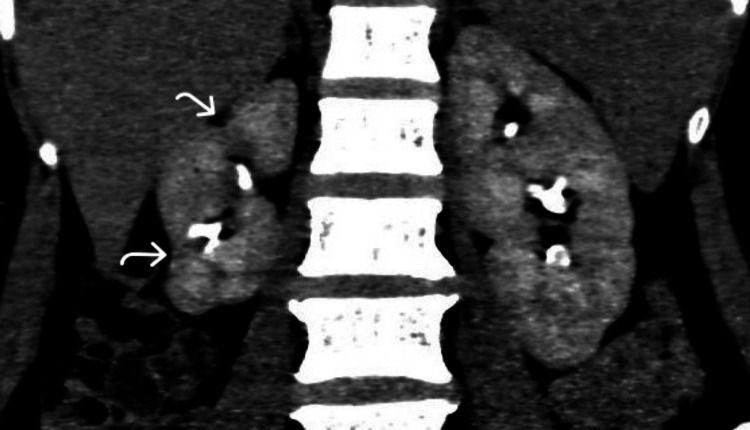
Chronic pyelonephritis. A 55-year-old woman with right loin pain was diagnosed with chronic pyelonephritis. Coronal reformatted image of delayed phase CT urogram demonstrates a shrunken right kidney with irregular contour and focal cortical scars (curved arrows) in the polar regions. These images are from the hospital and have not been published elsewhere. CT: computed tomography

The CT findings can be categorized into parenchymal and calyceal changes. Parenchymal changes include focal cortical scarring (see Figure 8) primarily affecting the poles, cortical thinning overlying deformed calyces, global atrophy with the loss of renal volume, and the compensatory hypertrophy of uninvolved segments or the contralateral kidney [24]. Calyceal changes include clubbing and dilatation, the blunting of calyceal fornices, and irregular calyceal contours [15]. Additional findings may include cortical calcifications, hydronephrosis if associated with obstruction, or parenchymal atrophy with the preservation of the renal hilum ("ask-in-kidney") [16].

Differential diagnosis for chronic pyelonephritis: Various conditions and normal variants can simulate chronic pyelonephritis on imaging studies, including fetal lobulations, renal infarcts, and reflux nephropathy. Fetal lobulations are characterized by smooth indentations located between calyces (not directly overlying them) with normal calyceal configuration and the absence of parenchymal scarring [5]. Renal infarcts manifest as wedge-shaped cortical scars with preserved calyceal structures. Unlike chronic pyelonephritis, they are not predominantly located in polar regions and may be accompanied by evidence of vascular pathology [24]. Reflux nephropathy shares imaging similarities with chronic pyelonephritis but has a specific association with vesicoureteral reflux, is more common in pediatric populations, and may be accompanied by additional ureteral abnormalities [15]. Differentiation among these entities relies on the careful assessment of imaging features and clinical context.

Complications in pyelonephritis

Pyonephrosis

Pyonephrosis is a urologic emergency referring to the presence of purulent material within an obstructed collecting system and requires prompt intervention [6]. Key CT findings include hydronephrosis with an identifiable cause of obstruction, increased density of urine in the collecting system due to the presence of purulent material (possibly with visible debris levels) creating heterogeneous fluid attenuation, and an enhancing, thickened renal pelvis wall indicating inflammation [9]. Distinguishing between simple hydronephrosis and pyonephrosis can be challenging on imaging alone, and clinical correlation may be necessary [6].

Renal and Perinephric Abscesses

**Figure 9:**
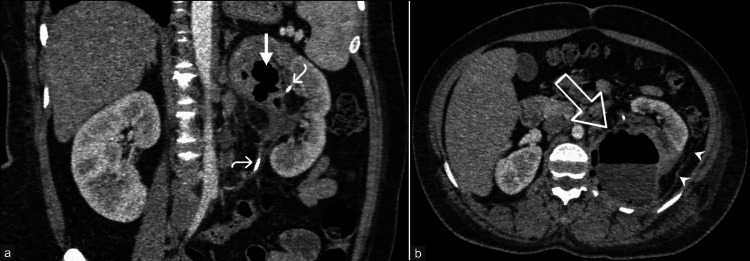
Complicated emphysematous pyelonephritis with renal abscess formation. A 38-year-old woman with a history of ureteroscopic lithotripsy and complaints of left loin pain was diagnosed with complicated acute emphysematous pyelonephritis. (a) Coronal reformatted image of nephrographic phase CT urogram shows relatively enlarged left kidney with architectural distortion, the heterogeneous enhancement of left renal parenchyma with reduced contrast uptake in the affected upper pole, and multiple air pockets (straight arrow) in the parenchyma and pelvicalyceal system. Portions of the proximal end of the double-J stent (curved arrows) can be seen in the left kidney. (b) Axial nephrographic phase CT image shows the presence of a well-defined collection (see-through arrow) epicentered in the upper pole of the left kidney, demonstrating air-fluid level and rim enhancement. The thickening and enhancement of the posterior renal fascia (arrowheads) is also seen. These images are from the hospital and have not been published elsewhere. CT: computed tomography

**Figure 10:**
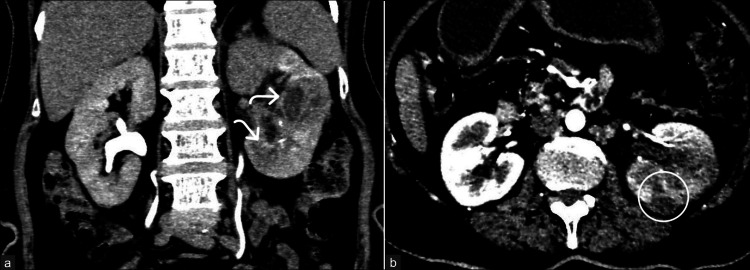
Acute pyelonephritis complicated by multifocal renal abscesses. A 55-year-old woman complaining of left loin pain and dysuria was diagnosed with acute pyelonephritis of the left kidney with multifocal renal abscesses. (a) Coronal reformatted image in the delayed phase of CT urogram shows relatively enlarged left kidney with edematous cortex and multifocal heterogeneous peripherally enhancing cystic areas (curved arrows), suggestive of abscesses. (b) Post-processing brightness-adjusted axial image in the delayed phase shows the rupture of one of the cystic lesions (circle) in the lower pole. These images are from the hospital and have not been published elsewhere. CT: computed tomography

Renal and perinephric abscesses represent serious complications of pyelonephritis, occurring when infection leads to liquefactive necrosis within the kidney or surrounding tissues [4]. The appearance of renal abscesses evolves over time, making contrast administration beneficial to characterize CT findings, which range from ill-defined areas of decreased enhancement in early stages to well-defined, intracapsular hypodense lesions with a thick, irregular wall demonstrating rim enhancement as the abscess matures [18]. Serial imaging may be necessary to monitor treatment response (see Figures 9, 10) [9]. A central fluid attenuation area indicative of necrosis, potential gas bubbles within the collection, and inflammatory changes in the adjacent parenchyma may also be observed [4]. A perinephric abscess should be suspected when there is a collection outside the renal capsule but within Gerota's fascia, with an enhancing rim or pseudo capsule causing mass effect on the kidney and the thickening of the perirenal fascia [18].

## Conclusions

Computed tomography has become an essential tool in the assessment of pyelonephritis and its various complications, significantly influencing clinical management decisions. While imaging is not typically required for straightforward cases of acute pyelonephritis, CT plays a crucial role in evaluating patients with risk factors for complications, those showing inadequate response to treatment, and situations where alternative diagnoses are being considered. The ability of a radiologist to recognize the specific imaging patterns of different types of pyelonephritis facilitates accurate diagnosis, appropriate treatment planning, and timely intervention when indicated. CT findings must be interpreted within the broader context of clinical presentation and laboratory findings for optimal diagnostic accuracy.

The development of reduced-dose CT protocols represents an important area of research to minimize radiation exposure while preserving diagnostic quality, particularly for patients requiring serial imaging examinations. Additionally, emerging technologies such as dual-energy CT hold promise for enhancing the characterization of renal infections and their complications.
